# Changes in Self-Regulation-Related Prefrontal Activities in Eating Disorders: A Near Infrared Spectroscopy Study

**DOI:** 10.1371/journal.pone.0059324

**Published:** 2013-03-19

**Authors:** Chihiro Sutoh, Michiko Nakazato, Daisuke Matsuzawa, Kadushi Tsuru, Tomihisa Niitsu, Masaomi Iyo, Eiji Shimizu

**Affiliations:** 1 Department of Cognitive Behavioral Physiology, Graduate School of Medicine, Chiba University Chiba Chuo, Chiba, Japan; 2 Research Center for Child Mental Development, Graduate School of Medicine, Chiba University Chiba Chuo, Chiba, Japan; 3 Department of Psychiatry, Graduate School of Medicine, Chiba University, Chiba Chuo, Chiba, Japan; 4 Department of Child Psychiatry, Chiba University Hospital, Chiba Chuo, Chiba, Japan; University of Medicine & Dentistry of NJ - New Jersey Medical School, United States of America

## Abstract

**Objective:**

The aim of this study is to clarify the symptomatology of the eating disorders examining the prefrontal function and activity associated with self-regulation among participants with or without eating disorders.

**Methods:**

Ten patients with anorexia nervosa, fourteen with bulimia nervosa, and fourteen healthy control participants performed two cognitive tasks assessing self-regulatory functions, an auditorily distracted word fluency task and a rock-paper-scissors task under the measurements on prefrontal oxyhemoglobin concentration with near infrared spectroscopy. The psychiatric symptoms of patient groups were assessed with several questionnaires.

**Results:**

Patients with bulimia nervosa showed decreased performances and prefrontal hyper activation patterns. Prefrontal activities showed a moderate negative correlation with task performances not in the patient groups but only in the healthy participants. The prefrontal activities of the patient groups showed positive correlations with some symptom scale aspects.

**Conclusions:**

The decreased cognitive abilities and characteristic prefrontal activation patterns associated with self-regulatory functions were shown in patients with bulimia nervosa, which correlated with their symptoms. These findings suggest inefficient prefrontal self-regulatory function of bulimia nervosa that associate with its symptoms.

## Introduction

Eating disorders (EDs) are life-threatening mental diseases that include anorexia nervosa (AN), bulimia nervosa (BN), and related disorders. ED patients take extreme preoccupation with foods and desire for thinness. They make an effort to achieve significant weight loss through restricting fattening food, exercising, and/or purging behaviors (self-induced vomiting, laxatives/diuretics abuse), but they sometimes overeat impulsively – bingeing.

Personality traits are important predisposition for ED. Both AN and BN patients are characterized by their personality traits, such as perfectionism, obsessive-compulsiveness, neuroticism, negative emotionality, harm avoidance, low self-directedness, and low cooperativeness [1], [2]. Patients with these features causing an impairment of daily life are diagnosed as having comorbid personality disorders, which can affect symptoms [3], dysregulation of dietary behavior [4], and the onset and course [5]–[7] of ED. One highlighted aspect is self-regulation with its potential contribution to the symptoms of ED. Changes in self-regulation have been discussed in the context of characteristic personality and pathologic behavior of ED, especially related with impulsivity. Binge eating/purging type of AN and BN show loss of self-control [1], [3], [8], [9] while restrictive type of AN accompanies overly controlled behavioral style [10], [11]. Binge eating/purging type of AN and BN, which have in common bulimic behaviors and sensitivity to substance use problems, are thus considered to be related to difficulties in self-regulation seen as an impulsivity [1], [12]. According to a review paper, changes in self-regulation seem relevant to deficits in the executive functioning such as set-shifting that is also observed in ED [13].

Abnormal prefrontal function of ED had been shown in previous investigations with functional neuroimaging methods. Typically, studies have used tasks/stimuli provoking core symptoms and shown abnormal activations of brain areas including the prefrontal cortex (PFC) [14]–[16]. These authors explained the observed involvement of prefrontal areas as representations of compulsive feature of behavior [14] and emotional processing and aversive response to the provocative stimuli [14], [17], [18]. Provocation studies with distorted body images induced incongruent results in the PFC activations of patient groups [19]–[21]. These authors explained such inconsistent decreased activities of patient groups associating with a realistic perception of own body size and shape [20], however possibly including influence of difference in stimuli [19], [21].

PFC is known to be involved in self-regulation of irrelevant behavior [22]–[26] and emotion [27]. The cognitive down-regulation of craving was associated with activity in prefrontal regions associated with regulating emotion and cognitive control in general, including dorsomedial, dorsolateral, and ventrolateral prefrontal cortices [28]. To examine associations between self-regulation, symptoms, and brain function of ED patients, researchers have used neuroimaging studies in combination with unique tasks that relate seemingly “indirectly” with symptoms. For instance, a monetary reward gambling task was used to reflect an improper value judgement of ED patients [29]. With the Simon spatial incompatibility task associated with self-regulatory control, BN patients showed an impaired activation pattern in their prefrontal neuronal network [30]. Despite the proposed importance to understand pathophysiology of characteristic self-regulation of ED, little has been shown addressing their relationships with prefrontal function.

The aim of the present study was to investigate prefrontal functions associated with self-regulation and symptoms of ED. We utilized two cognitive tasks related with self-regulation, an auditorily-distracted word fluency task (WFT) to assess inhibitory processing for emotional distraction, and a rock-paper-scissors task with intentional loss (RPST_loss_) to assess self-regulatory control on habitual behavior [23], [25], [31] and emotion. To assess the prefrontal activity evoked with the cognitive tasks, we measured prefrontal oxyhemoglobin concentrations ([oxyHb]) with near infrared spectroscopy (NIRS).

Our hypotheses were that (i) ED patients had changed cognitive abilities seen in the task performances associated with self-regulation; (ii) the expected abnormal cognitive functions were accompanied by changes in prefrontal blood perfusion; and (iii) the cognitive abnormalities and prefrontal activity were associated with the severity of ED symptoms measured with symptom scales.

## Methods

### Participants

Twenty-four female patients fulfilling the DSM-IV-TR criteria of EDs were recruited from the inpatient (N = 5) and outpatient (N = 19) units of Chiba University Hospital (Table 1) and divided into the AN group (10 patients; four restrictive type and six binge eating/purging type) and the BN group (14 patients; 13 purging type and one non-purging type). Among the patients with AN, two patients were medicated with antipsychotics (olanzapine and sulpiride). Among the patients with BN, three were medicated with antidepressants only (sertraline, setiptiline and lofepramine), one with both antidepressant (trazodone) and antipsychotics (olanzapine and risperidone), and ten without either antidepressants or antipsychotics. Anxiolytics, hypnotics, and tranquilizing drugs were prescribed for two patients with AN and six patients with BN. One patient with AN had comorbid major depression and another with AN had dissociative disorder, and no other participant was diagnosed as having comorbid psychiatric disorder. Three patients classified into the BN group had past history of AN. Fourteen age-matched healthy control (HC) females who had no history of psychiatric disorder were also recruited. Exclusion criteria for the participants were left-handed, comorbid progressive physical disorder, and a history of personality disorder, head injury, drug dependency, or alcoholic dependency. All participants were native Japanese and very familiar with the ordinary rock-paper-scissors game for a long time since a young age. All subjects participated after written informed consent for study participation was obtained. The study was approved by the Institutional Review Board of the Graduate School of Medicine, Chiba University and conformed to the provisions of the Declaration of Helsinki in 1995.

**Table 1:** Demographics of all participants, and scores of symptom scales relevant to the ED group.

				HC (N = 14)		ED (total N = 24)									η^2^
						Total			AN (N = 10)			BN (N = 14)			
				mean	SD	mean	SD	N	mean	SD	N	mean	SD	N	
Age				24.1	3.0	26.1	7.1	24	27.7	8.4	10	25.0	6.1	14	0.06
Height				159.7	4.9	157.4	4.6	23	157.1	4.7	10	157.7	4.7	13	0.06
Weight				53.7	7.5	45.8	8.2	23	42.2	8.7	10**	48.6	6.9	13	0.28
BMI				21.0	2.2	18.5	3.2	23	17.0	3.1	10**	19.6	3.0	13	0.27
Period of illness (months)						67.6	53.5	21	42.7	34.6	7	80.1	57.9	14	
Treatment period (months)						36.6	42.9	20	19.4	16.8	7	45.9	50.1	13	
Antidepressants treatment								4			0			4	
Antipsychotics treatment								3			2			1	
Anxiolytics treatment								8			2			6	
History of hospitalization						(11 of 20 patients had)			(5 of 7 patients had)			(6 of 13 patients had)			
HADS			Anxiety	3.3	1.9	11.1	3.0	24	10.8	3.0	10***	11.4	3.1	14***	0.69
			Depression	1.8	1.6	9.3	3.7	24	6.8	3.4	10***	11.1	2.8	14***	0.72
EDI						127.9	41.4	15	107.3	42.4	6	141.6	36.8	9	
EDE-Q			Global			3.9	1.3	16	3.3	1.5	6	4.2	1.1	10	
			Restraint			3.3	1.7	16	3.4	1.8	6	3.2	1.6	10	
			Eating			3.9	1.5	17	3.4	1.8	7	4.2	1.4	10	
			Weight			4.2	1.6	17	3.4	1.7	7	4.7	1.3	10	
			Shape			4.4	1.3	16	3.8	1.6	6	4.7	1.1	10	
BITE			Severity			13.4	5.8	13	12.0	7.1	4	14.0	5.5	9	
			Symptom			22.9	3.5	13	22.0	5.3	4	23.3	2.6	9	
TAS-20						65.4	5.2	15	63.4	3.5	5	66.4	5.7	10	

Notes: One-way analysis of variances was performed to compare age, height, weight, BMI and HADS subscales between the HC, AN and BN groups. HC, healthy control; ED, eating disorder; AN, anorexia nervosa; BN bulimia nervosa; SD, standard deviation; BMI, body mass index; HADS, Hospital Anxiety and Depression Scale; EDI, the Eating Disorder Inventory; EDE-Q, the Eating Disorder Examination Questionnaire, BITE, the Bulimic Investigatory Test; TAS-20, the 20-item Toronto Alexithymia Scale. Significant differences between each patient group with the HC group were shown with ***p<0.001,

** *p*<0.01 or *p<0.05.

### Cognitive Tasks

The cognitive tasks to evoke prefrontal cortical activity, the WFT and the RPST_loss_, were performed by all participants. The subjects sat on a comfortable chair in a room lit by daylight. The tasks were computed with RSVP Experiment ver. 1.1 [32]. Subjects were visually instructed regarding the performance of all task procedures and were cued on a PC display placed in front of them.

#### 1) WFT

**Figure 1:**
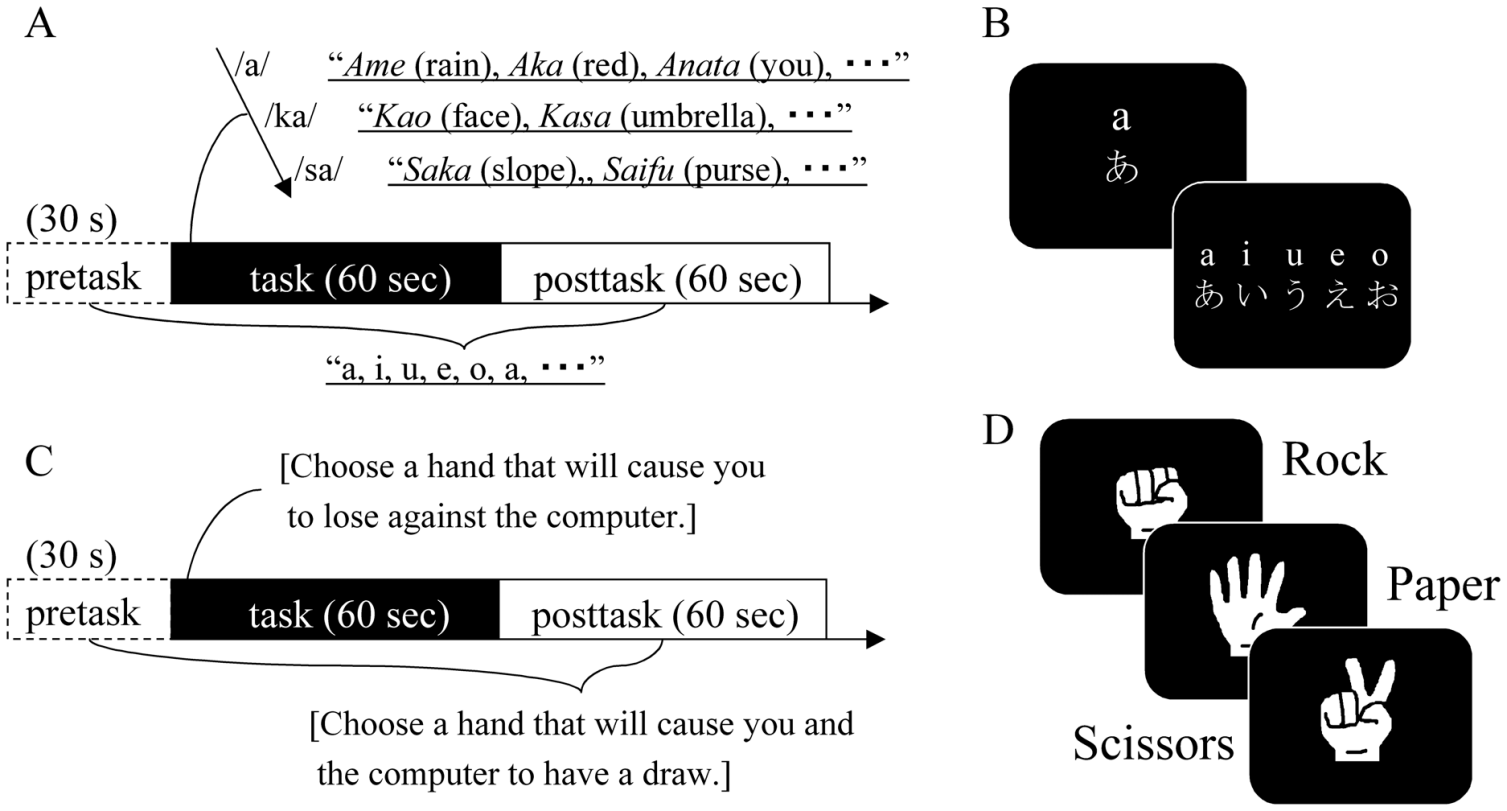
Task procedures of the word fluency task (WFT) and the rock-paper-scissors intentional loss task (RPST_loss_). (A) Time schedule of one trial of the WFT. (B) Visual instructions presented in the WFT. A set of three different syllables was presented for 20 sec each during a 60-sec task period, to serve as the initial syllables for word generation. During the pretask and posttask periods, participants were instructed to repeat the presented five syllables in turn. (C) Time schedule and instructions of one trial of the RPST_loss_. (D) Three hand shape pictures presented in the RPST_loss_. The same pictures were used during the pretask, task, and posttask periods.

In the previous researches, WFT has been used as “a general frontal activating task” that focus on evidence of neural functions (e.g., activating areas) and experimental usability than on relations with traits or symptoms [33], [34]. We modified it aiming at exposing emotion inhibition adding auditory distracters during performing ordinary WFT. The subjects performed two WFT trials, each consisting of a 30-sec pretask baseline, a 60-sec task, and a 60-sec posttask (Figure 1A). During task periods of the WFT, the subjects were instructed to generate as many words as they could that had a particular initial syllable [34]. There were two syllable-sets prepared to be presented: (A)/a/,/ka/, and/sa/, and (B)/i/,/ki/, and/shi/. In each 60-sec task period, the three syllables within one of the two syllable-sets were presented, changing in turn every 20 sec (Figure 1B). The number of words generated during the task was determined as a task performance. In pretask and posttask periods, the subjects were instructed to repeat the syllables,/a/,/i/,/u/,/e/, and/o/at approximately 1 Hz.

During each WFT trial, one of two auditory distracters, baby crying or white noise; was presented with a headphone at approximately 70 dB(A) at peaks. Baby crying was expected to be a stronger distracter than white noise, to arouse not only auditory neuronal processing but also emotion-inhibitory processing. Baby crying was also expected as a non-verbal noise that reduces the risk of giving participants any verbal “cues” to answer WFT [35]–[37]. The two trials were separated by an interval of at least 2 min. The presenting order of stimuli consisting of two syllable-sets and two distracters was determined in a pseudorandom order among the participants.

#### 2) RPST_loss_

Use of the ordinary rock-paper-scissors game is a simple and easy method to divide two or more players into winner and loser, and it is more familiar and played more frequently than a coin toss for most Japanese. Players inwardly choose one of three hand shapes, a fist for a rock, a flat palm for a piece of paper, or splayed second and third fingers for scissors, and then simultaneously present their hands while forming the chosen shapes. When two players participate, the relationship between the hands of the two players decides their outcomes of winning, losing, or having a draw. Rock beats scissors because a rock break metal, paper beats rock because paper wraps around a rock, and scissors beats paper because scissors cut a paper. If the same hand shapes are presented, the result is a draw and the game is replayed until a player wins.

In order to assess self-regulatory control, we developed the RPST_loss_ (Figure 1C) based on an ordinary rock-paper-scissors game. In this task, the three pictures of hand shapes were shown one by one in a random order (Figure 1D). The subjects were instructed to respond by hitting any one of three buttons corresponding to each of three hand shapes, at which point the picture disappeared and the next picture was shown after 300 ms of a blank display. The subjects were told to respond so that they would intentionally lose the games during a task period and so that they would reach a draw during pretask and posttask periods as fast and correctly as possible [31]. Each of such three periods lasted until it met predefined requirements for both time and the number of games played, >30 sec and >30 games for the pretask, >60 sec and >48 games for the task, and >60 sec and >60 games for the posttask. For these numbers of games (30, 48, and 60 games), the ratio of correct responses ("accuracy") and the mean reaction time were determined as the subjects’ performances. To analyze the [oxyHb] data of RPST_loss_, the time lengths of which varied with the individual participants, 30 sec of initial pretask period, 60 sec of initial task period, and 60 sec of initial posttask period were extracted to consecutively combined. Hence, the time periods used for the analyses of [oxyHb] overlapped but were strictly incongruent with those for the performances.

### NIRS Measurements

**Figure 2:**
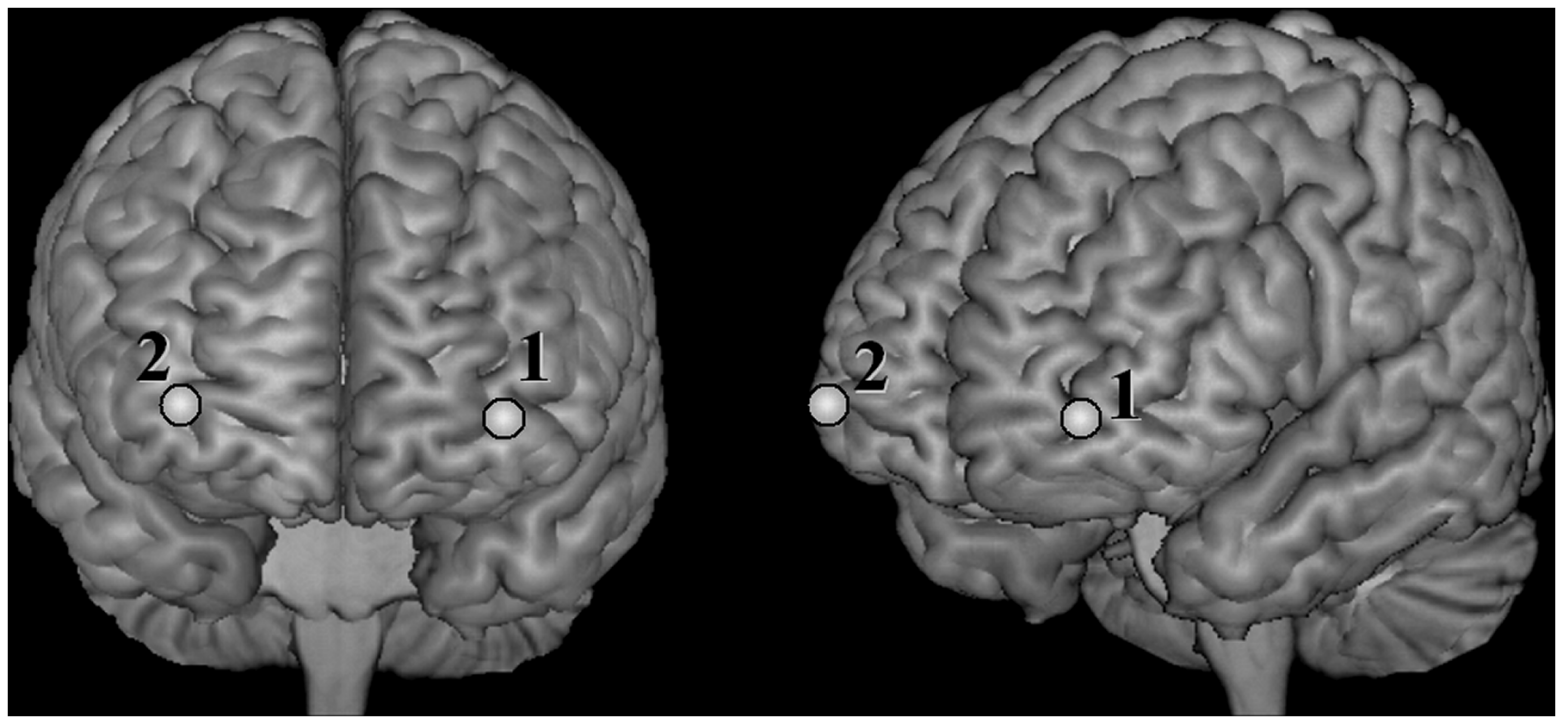
Brain sites observed with near infrared spectroscopy. The oxyhemoglobin concentrations in the left and right regions (channels 1 and 2, respectively) over the lower dorsolateral prefrontal to orbitofrontal areas were measured. These regions are labelled in the standard brain space [38], [39].

During the tasks, prefrontal [oxyHb] changes were measured with two-channel NIRS (NIRO-200, Hamamatsu Photonics K.K., Hamamatsu, Japan) at three wavelengths of near infrared light (775, 810 and 850 nm) with a sampling rate of 2 Hz. The detection probes were placed on the Fp1 and Fp2, and the emission probes were 3 cm lateral from the detection probes along the T3-Fpz-T4 line, according to the international 10/20 system used in electroencephalography. The points measured with this setup were projected to the lower dorsolateral PFC to the orbitofrontal areas (BA 9–10, Figure 2) [38], [39].

The acquired [oxyHb] data were preprocessed as described below. Five-sec moving average methods were done to attenuate high-frequency components such as motion artifacts. Data were transformed into the z score against the mean [oxyHb] of the pretask period in each channel. Linear data-trends between the mean of a pretask period and the last twenty sec of a posttask period were removed from the z scores of the WFT to remove a low-frequency band as noise. Removal of linear data-trend was not performed on data of the RPST_loss_ to avoid removal of different frequency band by the individual participants, because of varying time length spent for this task. The z scores of task and posttask periods were each divided into first and last halves (task1, task2, posttask1, and posttask2), averaged respectively, and subjected to statistic analyses.

### Assessments of Clinical Symptoms

All participants completed the Hospital Anxiety and Depression Scale (HADS) [40], and ED group were assessed using the Eating Disorder Inventory-3 (EDI) [41], the Eating Disorder Examination Questionnaire (EDE-Q) [42], the Bulimic Investigatory Test, Edinburgh (BITE) [43], and the 20-item Toronto Alexithymia Scale (TAS-20) [44], [45].

### Statistical Analyses

The experimental values were statistically analyzed with SPSS 12.0 (SPSS, Inc., Chicago, IL). One-way analysis of variances and Bonferroni’s comparisons were performed for the variables measured in all participant groups, that is, demographics (age, height, weight and BMI) and scores of symptom scales (two subscales of HADS). To extenuate possible effects of malnutrition for cognitive functioning (e.g., effects of cortisol [46] and Triiodothyronine [47]), the analyses for task performances were done controlling for BMI as a covariate of no interest only if these parameters met prerequisites to be controlled; 1) showing no significant interaction with BMI and 2) showing significant linear correlation with BMI. Consequently, no task performance parameter was controlled for BMI. Repeated measures of analysis of variances and Bonferroni’s comparisons were performed for task performances and [oxyHb] data. Exploratory correlation analyses were performed between [oxyHb] data, task performances, and symptom scales. As correlation coefficients we used Spearman's rho for data pair including one non-normal variable (or both) and Pearson's *r* for data pairs with only normal variables. Bonferroni’s correction was not performed for the probabilities of correlation coefficients in order to avoid attenuating power of detection in this study. Values with *p*<0.05 were considered statistically significant.

## Results

### Demographics and Clinical Characteristics

The demographics and scores of symptom scales of participants are listed in Table 1. The AN, BN, and also the group with combined ED showed elevated scores in both HADS subscales compared to the HC.

### WFT (Figures 3, 4)

**Figure 3:**
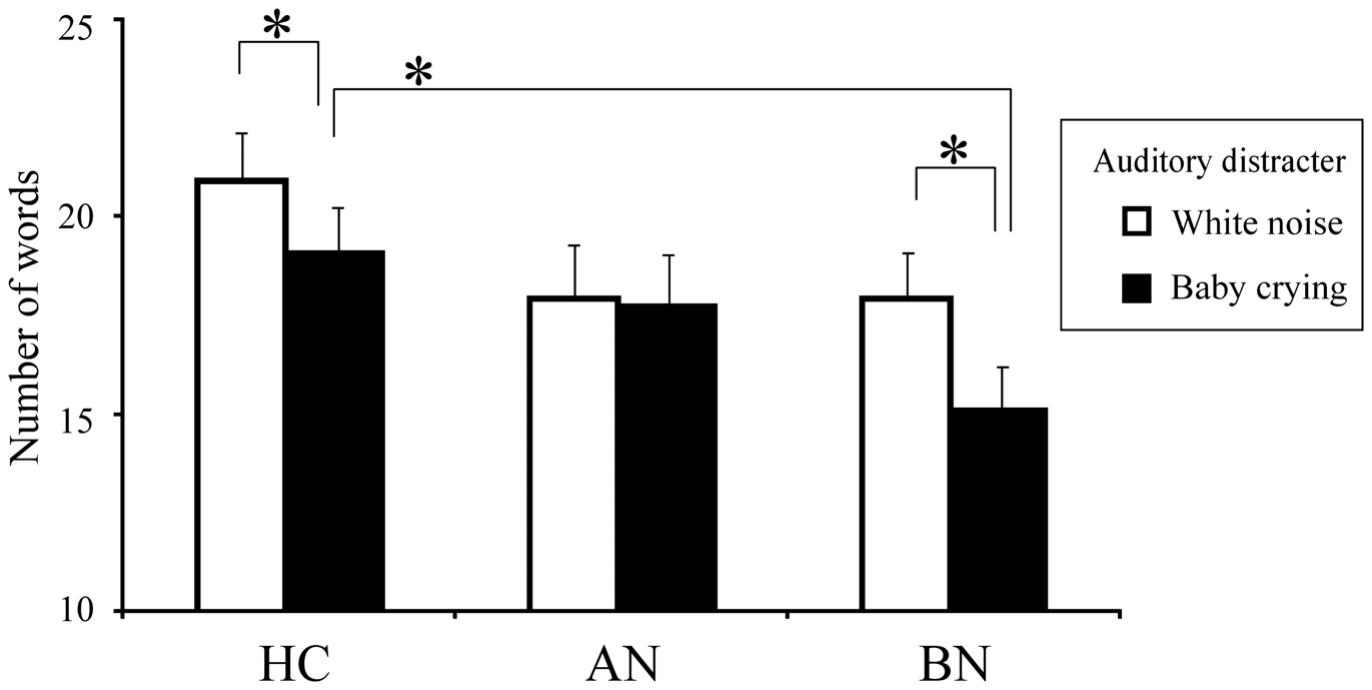
Mean numbers of generated words during task periods of the word fluency task (WFT). Two WFT trials were performed with two types of auditory distracters, respectively: white noise (outlined bars) and baby crying (filled bars). Baby crying significantly affected word generation by the HC and BN groups. The BN group generated a significantly lower number of words than the HC when during hearing the crying baby distracter. Error bars represent the standard error of the mean. HC, healthy control; AN, anorexia nervosa; BN, bulimia nervosa; *p<0.05.

**Figure 4:**
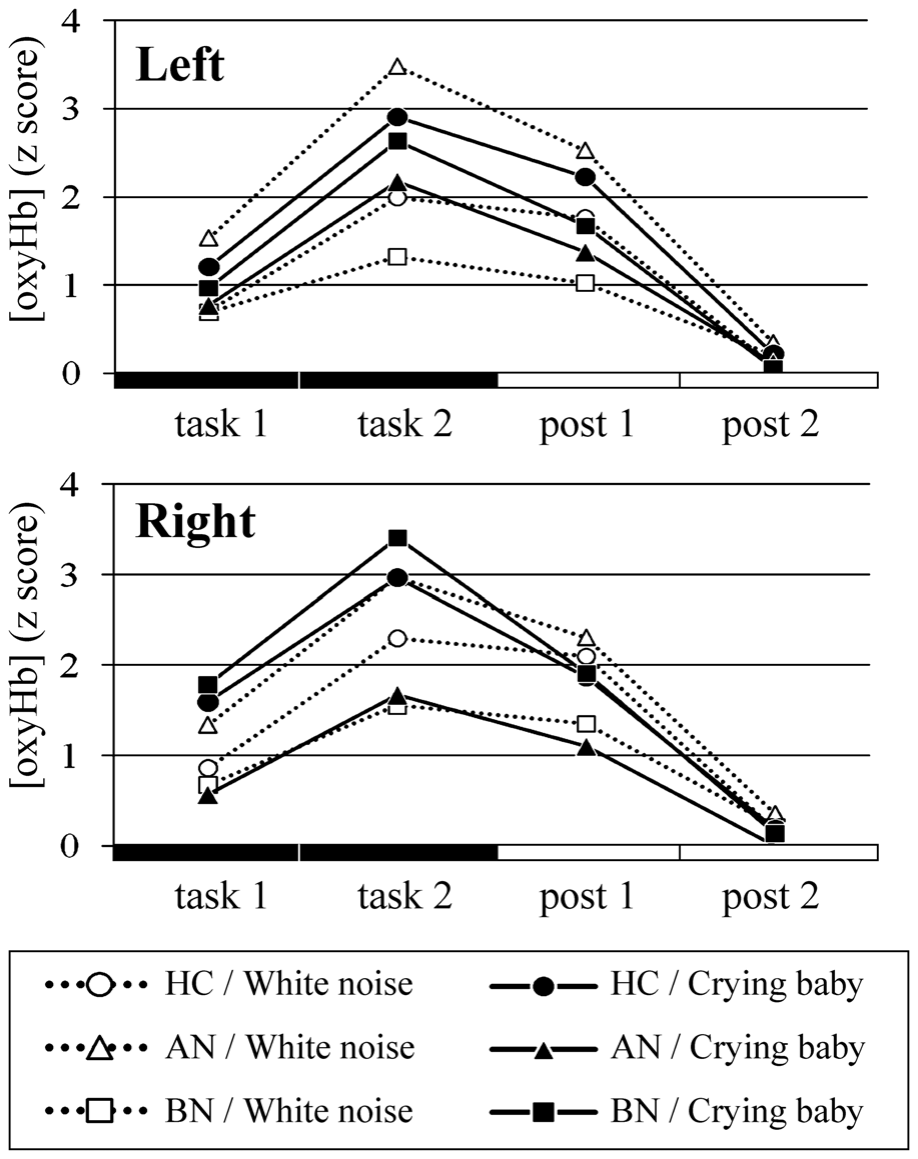
Oxyhemoglobin concentration changes during the word fluency task in the bilateral prefrontal cortices. Mean z scores of [oxyHb] during the task and posttask periods each divided into first and last halves are shown. Circular, triangular, and square plots represent the healthy control, anorexia nervosa, and bulimia nervosa groups, respectively. Outlined plots with dashed lines and filled plots with solid lines represent [oxyHb] with white noise and baby crying as an auditory distracter, respectively. There was no significant difference between groups or between auditory distracters. HC, healthy control; AN, anorexia nervosa; BN, bulimia nervosa.

Regarding the number of words produced during the WFT, a significant interaction was observed between the group factor and the distracter factor (*F*(2,34) = 3.389, *p* = 0.045, partial η^2^ = 0.166). Simple main effect of the group factor during hearing baby crying (*F*(2,34) = 3.750, *p* = 0.034, partial η^2^ = 0.181) suggested that the BN group generated significantly fewer words than did the HC (*p* = 0.033) and no difference was seen between the AN group and the HC during the task (*p* = 1.000). A significant main effect of the auditory distracter factor was seen (*F*(1,34) = 15.900, *p*<0.001, partial η^2^ = 0.319), as expected. Comparing the two auditory distracters, baby crying was found to affect word generation significantly than white noise in the patients with HC (*F*(1,34) = 7.277, *p* = 0.011, partial η^2^ = 0.176) and BN (*F*(1,34) = 18.771, *p*<0.001, partial η^2^ = 0.356), whereas the AN group showed no significant difference (*F*(1,34) = 0.066, *p* = 0.799, partial η^2^ = 0.002). This analysis was performed not controlled for BMI because there was no significant linear correlation (with white noise, *t* = 0.817, *p* = 0.420; with baby crying, *t* = −0.438, *p* = 0.664) between BMI and the distracter factor, while there was a significant interaction (*F*(1,33) = 4.564, *p* = 0.040).

The [oxyHb] changes in bilateral prefrontal regions during the WFT depicted a regular arching pattern within all groups and conditions commonly, which increased during the task and decreased during the posttask period. Among the auditory distracter factor, the task phase factor, and the group factor, there was a significant secondary interactions in the left (*F*(6,102) = 2.331, *p* = 0.038, partial η^2^ = 0.118) but right (*F*(6,102) = 1.468, *p* = 0.197, partial η^2^ = 0.079) channels. However, no significant intergroup difference was found in *post hoc* multiple comparisons bilaterally. There was no significant primary interaction [left, (group*distracter, *F*(2,34) = 3.129, *p* = 0.057, partial η^2^ = 0.155; group*task phase, *F*(6,102) = 0.417, *p* = 0.866, partial η^2^ = 0.024; distracter*task phase, *F*(3,102) = 0.702, *p* = 0.553, partial η^2^ = 0.020); right, (group*distracter, *F*(2,34) = 2.314, *p* = 0.114, partial η^2^ = 0.120; group*task phase, *F*(6,102) = 0.090, *p* = 0.997, partial η^2^ = 0.005; distracter*task phase, *F*(3,102) = 1.564, *p* = 0.203, partial η^2^ = 0.044)]. A significant main effect of the task phase factor was detected (left, *F*(3,102) = 30.560, *p*<0.001, partial η^2^ = 0.473; right, *F*(3,102) = 20.918, *p*<0.001, partial η^2^ = 0.381) and the [oxyHb] differed between all four time points. No significant main effect of the auditory distracter factor (left, *F*(1,34) = 0.046, *p* = 0.831, partial η^2^ = 0.001; right, *F*(1,34) = 0.062, *p* = 0.805, partial η^2^ = 0.002) or the group factor (left, *F*(2,34) = 0.365, *p* = 0.697, partial η^2^ = 0.021; right, *F*(2,34) = 0.051, *p* = 0.950, partial η^2^ = 0.003) was seen in the bilateral channels. In each patient group, classifying and comparing the [oxyHb] data of participants with or without each type of drug medication suggested that medication did not contribute our findings.

### RPST_loss_ (Figures 5, 6)

During interviews after the examinations, almost all of the participants reported that intentionally losing the games was more difficult than playing to a draw and that they felt chagrined at erroneously winning. Regarding the accuracy of responses, there were no significant interaction (*F*(4,64) = 0.981, *p* = 0.424, partial η^2^ = 0.056) or main effect found among the task phase factor (*F*(2,66) = 0.830, *p* = 0.441, partial η^2^ = 0.025) and the group factor (*F*(2,33) = 0.698, *p* = 0.505, partial η^2^ = 041). No difference was observed between groups in *post hoc* comparisons. Analyses on the reaction time in RPST_loss_ suggested that the BN group had a longer reaction time than that of the HC during the task period (*F*(2,33) = 4.165, *p* = 0.024, partial η^2^ = 0.202), even though no significant interaction was detected between the group factor and the task phase factor (*F*(4,64) = 2.186, *p* = 0.090, partial η^2^ = 0.117). The main effects of the group factor (*F*(2,33) = 4.167, *p* = 0.024, partial η^2^ = 202) and the task phase factor (*F*(2,66) = 106.079, *p*<0.001, partial η^2^ = 0.763) were found. The accuracy variable was not controlled for BMI because BMI showed no significant interaction (*F*(2,64) = 0.008, *p* = 0.992) or a significant linear correlation (the pretask, *t* = 1.237, *p* = 0.225; the task, *t* = 0.687, *p* = 0.497; the posttask, *t* = 0.351, *p* = 0.728) with the task phase factor. The reaction time variable was also not controlled for BMI because of no significant interaction (*F*(2,64) = 0.001, *p* = 0.999) or a significant linear correlation (the pretask, *t* = −1.774, *p* = 0.086; the task, *t* = −0.590, *p* = 0.559; the posttask, *t* = −0.908, *p* = 0.371) between BMI and the task phase factor.

**Figure 5:**
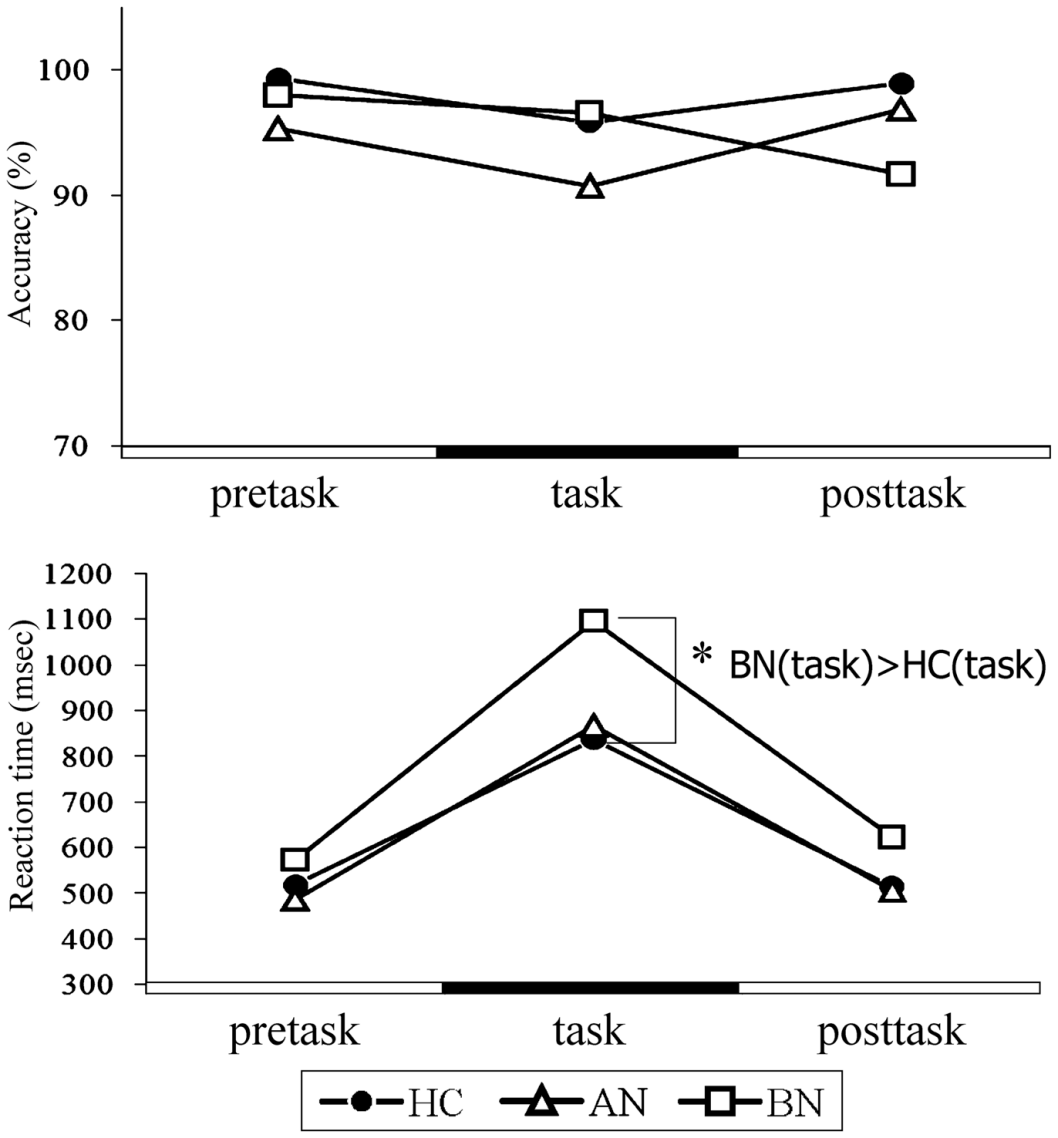
Mean values of accuracy and reaction time of the rock-paper-scissors intentional loss task. Mean values of accuracy and reaction time of pretask and posttask (instructed to be draw games) and task (instructed to lose intentionally) periods are shown. Circles, triangles, and squares represent the healthy control, anorexia nervosa, and bulimia nervosa groups, respectively. There was no group difference in accuracy. The bulimia group showed a significantly longer reaction time than that of the healthy controls during the task period. BN, bulimia nervosa; HC, healthy control; *p<0.05.

**Figure 6:**
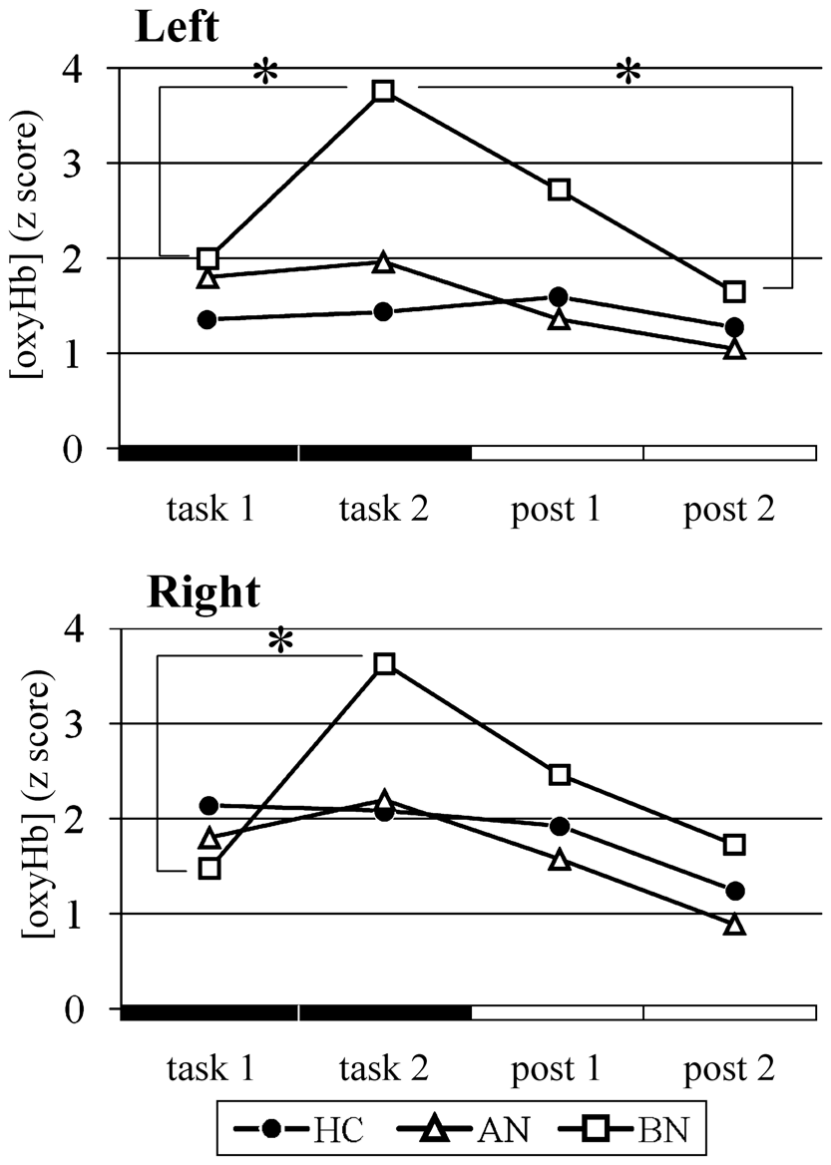
Oxyhemoglobin concentration changes during the rock-paper-scissors intentional loss task in the bilateral prefrontal cortices. Mean z scores of [oxyHb] during the task and posttask periods each divided into first and last halves are shown. Circles, triangles, and squares represent the healthy control, anorexia nervosa, and bulimia nervosa groups, respectively. Only the bulimia group showed increasing and decreasing activation patterns of [oxyHb]. *p<0.05.

The [oxyHb] changes during the RPST_loss_ were analyzed using the task phase factor and the group factor. In the [oxyHb] data, no interaction between the two factors (left, *F*(6,102) = 1.557, *p* = 0.167, partial η^2^ = 0.084; right, *F*(6,102) = 1.185, *p* = 0.320, partial η^2^ = 0.065) or a main effect of group factor (left, *F*(2,34) = 1.007, *p* = 0.376, partial η^2^ = 0.056; right, *F*(2,34) = 0.376, *p* = 0.690, partial η^2^ = 0.022) was observed. Multiple comparisons using both the task phase and group factors revealed a characteristic [oxyHb] change pattern in the BN group which is an increasing and decreasing activation pattern linked to the task course (left, *F*(3,32) = 7.053, *p*<0.001, partial η^2^ = 0.398; right, *F*(3,32) = 8.240, *p*<0.001, partial η^2^ = 0.436). The [oxyHb] of the BN group showed a significant elevation (left, *p* = 0.004; right, *p*<0.001) toward a peak at the latter half of the task period, and then a decrease (left, *p* = 0.008; right, *p* = 0.062; between the peak and the latter half of the posttask period). At this peak period, but not at other three periods, weak group difference of distribution was suggested in the left channel (left, *F*(2,34) = 2.547, *p* = 0.093, partial η^2^ = 0.130; right, *F*(2,34) = 1.592, *p* = 0.218, partial η^2^ = 0.086). Such activation pattern was not detected in the AN group (left, *F*(3,32) = 0.531, *p* = 0.664, partial η^2^ = 0.047; right, *F*(3,32) = 0.940, *p* = 0.433, partial η^2^ = 0.081) and the HC group (left, *F*(3,32) = 0.237, *p* = 0.870, partial η^2^ = 0.022; right, *F*(3,32) = 0.817, *p* = 0.494, partial η^2^ = 0.071). The patterns of the [oxyHb] changes associated with the RPST_loss_ were relatively flat, unlike those associated with the WFT, because of different preprocessing [oxyHb] data for each task (see 2.3 NIRS measurements). In each patient group, classifying and comparing the [oxyHb] data of participants with or without each type of drug medication suggested that medication did not contribute to our findings.

### Correlation Coefficients of Prefrontal Activity with Task Performances and Symptom Scales (Table 2)

To investigate the relationships of prefrontal activity with cognitive functions and symptoms in ED patients, we assessed the correlation coefficients between [oxyHb] data, task performance, and symptom ratings. Although no significant main group effect was pointed in any [oxyHb] data, we took notice of data at the latter half of the task period of RPST_loss_ for correlation analyses according to a result of a weak group difference of distribution suggested, because it showed characteristic values that could have different features between groups and be a biomarker of ED. At this phase the [oxyHb] data of only the HC showed a moderate negative correlation with the accuracy (left, Spearman’s rho = −0.548, *p* = 0.065; right, rho = −0.354, *p* = 0.259), while did not those of the AN group (left, rho = −0.031, *p* = 0.933; right, rho = 0.117, *p* = 0.747) and the BN group (left, rho = 0.297, *p* = 0.324; right, rho = −0.014, *p* = 0.963). No significant correlation was found between task performance variables and the [oxyHb] data at other task phases.

In the patient groups, the [oxyHb] data positively correlated with some ED symptom scales (Table 2). The left channel [oxyHb] data of the AN group positively correlated with the severity subscale of BITE (Pearson’s *r* = 0.977, *p* = 0.023). The [oxyHb] of the BN group correlated significantly positively with the global score (right; *r* = 0.690, *p* = 0.027) and the restraint subscale (left, *r* = 0.675, *p* = 0.032; right, *r* = 0.896, *p*<0.001) of EDE-Q, and the severity subscale (right, *r* = 0.811, *p* = 0.008) of BITE.

**Table 2:** Correlation coefficients of prefrontal activity with task performances and symptom scales.

		AN				BN			
		left		right		Left		Right	
		*r*	*p*	*R*	*p*	*R*	*p*	*r*	*p*
HADS	Anxiety	0.400	0.253	0.177	0.625	−0.267	0.357	−0.187	0.522
	Depression	0.465	0.176	0.461	0.180	−0.049	0.868	0.080	0.786
EDI		−0.040	0.940	−0.188	0.721	0.054	0.890	0.619	0.075
EDE-Q	Global	−0.377	0.462	−0.415	0.413	0.210	0.561	0.690	0.027*
	Restraint	−0.190	0.718	−0.442	0.380	0.675	0.032*	0.896	0.000*
	Eating concern	−0.337	0.460	−0.403	0.371	−0.109	0.764	0.385	0.271
	Weight concern	−0.249	0.590	−0.213	0.647	0.019	0.959	0.490	0.150
	Shape concern	−0.371	0.468	−0.389	0.446	−0.062	0.865	0.310	0.383
BITE	severity	0.977	0.023*	0.810	0.190	0.632	0.068	0.811	0.008*
	symptom	−0.494	0.506	−0.695	0.305	0.342	0.368	0.662	0.052
TAS-20		−0.541	0.347	0.101	0.872	−0.263	0.463	−0.005	0.990
BMI		−0.432	0.213	−0.209	0.563	−0.156	0.610	−0.329	0.273

Notes: Correlation coefficients were calculated between the symptom scales and mean z scores of [oxyHb] data during the latter half of the task period of the RPST_loss_. AN, anorexia nervosa; BN, bulimia nervosa; HADS, Hospital Anxiety and Depression Scale; EDI, the Eating Disorder Inventory; EDE-Q, the Eating Disorder Examination Questionnaire; BITE, the Bulimic Investigatory Test; TAS-20, the 20-item Toronto Alexithymia Scale; BMI, body mass index. *r*: Pearson’s correlation coefficient, *p<0.05.

## Discussion

Different profiles in the tasks associated with self-regulation were found between the BN group and the HC group: the BN group showed decreased cognitive functions in the WFT through the emotion inhibition task and the RPST_loss_, and the latter was accompanied by characteristic prefrontal activity. Moreover, prefrontal activity seen in the RPST_loss_ correlated with symptom scales in both the AN and BN groups. The data supports our first hypothesis that ED patients had changed cognitive abilities seen in the task performances associated with self-regulation.

### WFT

In the WFT, the BN patients hearing a baby crying showed lower verbal fluency than that of the HC, suggesting disordered emotion inhibition in the BN group. Previous research suggested that verbal fluency of ED patients did not differ from that of the HC but reflected frontal lobe dysfunction and ED symptoms [33]. We were able to measure the difference in verbal fluency between the groups that was not observed previously, probably because of using an additional factor, an auditory distracter. Neuropsychological studies have revealed various aspects of cognitive deficits in BN patients, such as executive functioning, visual-spatial ability, divided and sustained attention, verbal functioning, learning, and memory [13], [48], [49]. The decreased verbal fluency under existence of emotional auditory distracter in the BN group observed in this study should be a newly revealed aspect of this multidimensional cognitive impairment. This vulnerability of BN might reflect strong perceived expressed emotion [50] or distress tolerance which was associated with eating attitudes [51]. Meanwhile, the AN group showed no evidence of cognitive impairment, but another feature of the AN was a lack of auditory distracting effect expected, i.e., both sounds had an equal distractive effect. Since there is no difference in TAS-20 between the AN and BN groups, the effect of alexithymia on processing of both distracters was inapparent.

To our knowledge, this was the first study to measure prefrontal function measured with baby crying stimuli in the patients with ED. Prefrontal blood flow showed strong peaks reflecting the time course of the WFT. Unlike the result of task performance, no group difference was clarified in prefrontal activation. Word generation performed in the WFT is known to involve widespread brain areas including inferior frontal gyrus, medial frontal, supplementary motor area, premotor area, cingulate cortex, putamen, etc. [52]–[56]. Because of innate limitation of NIRS, [oxyHb] observation is limited to narrow and superficial regions. Taken together, brain areas other than those measured with NIRS but relating to word generation and/or emotional self-regulation might be involved with the decreased verbal fluency in the BN group only under an emotional auditory distracter. These results could propose a prudent view to usage of WFT just as “a common prefrontal activation task”, not as a task of verbal fluency. Further studies using techniques such as functional magnetic resonance imaging and positron emission tomography are required to investigate the involvement of other cortical areas than PFC as well as deep brain structures.

### RPST_loss_

The longer reaction time during the task period than during the pretask and posttask periods and impressions by participants suggest that it was more difficult to lose than to be draw for subjects who were familiar with an ordinary rock-paper-scissors game. In previous studies, the authors believed that the difficulty of intentionally losing reflected a malfunction of stereotypical response inhibition, since wanting to win the game is the stereotypical response [23], [25], [31]. The authors of these studies using the game have interpreted intentional loss trials as triggers of cognitive conflict and self-regulatory control associated with habitual behavior. Moreover, most participants reported self-accusation and feelings of inefficiency as part of their introspections associated with erroneous winning. In addition to the act of trying to win being habitual behavior, we propose that the self-regulation was also directed to emotions such as self-accusation, unpleasantness, or anger; i.e., reactions to perfectionistic ideas. Hence, the most likely reason for the prolonged reaction time during the task period compared to the pretask/posttask periods was the requirement for additional information processing within the brain.

The BN patients showed two specific features revealed with the RPST_loss_. First, they required a prolonged reaction time compared to that of the HC only during the task period. We propose that, applying the abovementioned concepts, BN patients had more difficulty self-regulating and required more effort to achieve self-regulation to achieve an equal level of accuracy to that of HC during task periods. Adversely, since no group difference was seen during the pretask and posttask periods, no difference was demonstrated in brain functions when playing to a draw, such as cognition of presented hand shapes, decision of the same hand shape, and choice and hitting the appropriate buttons.

Secondly, only the BN group had the increasing and decreasing [oxyHb] change pattern in PFC linked to the task course. Why were the prefrontal areas activated, and why in the BN group? The cognitive concept tested in the RPST_loss_ was self-regulation on habitual stereotypical behavior [23], [25]. Greater impulsivity and disinhibition (lack of control) are often seen in BN patients compared with AN patients as episodes of eating behaviors [3], [8], [9], and the DSM-IV diagnostic description of BN requires disinhibition over binge eating episodes. Moreover, PFC carries self-regulation of irrelevant behavioral responses [22], [24], [26], habitual stereotypical behavior [23], [25], and also emotion [27] by exerting an inhibitory physiological influence over other brain areas. A neuroimaging study showed that adolescent ED patients with binge eating/purging had greater activation during a response inhibition task in hypothalamus and right dorsolateral PFC compared to both HC and patients with restrictive type of AN [57]. In line with this context, we propose that the BN patients have lower and inefficient self-regulatory function of PFC that consequently appears as impulsivity. The term “inefficient” was derived from that, despite blunted functioning of PFC, the BN patients might require strong prefrontal activation to perform a difficult “to lose” task with the high accuracy equal to that of the HC. The prefrontal hyperactivity pattern of these patients might represent compensatory activation, i.e., the recruitment of additional brain regions, and/or discrepant brain activation patterns with impaired cognitive ability [58].

Bingeing has been considered to be related to impulsivity [1]. Patients with restrictive type of AN, i.e., without bingeing, are less impulsive than are HC [10], [11]. Indeed, in the present study, if the six binge eating/purging type of AN patients and all BN patients were combined into a “bingeing group”, their combined [oxyHb] data had a similar activation pattern to that of the original BN group, and the task performance variables (accuracy and reaction time) had no group difference (data not shown) supposedly because of limited participation of patients with restrictive type of AN (N = 4), following the findings in the previous study [57]. These results suggest that BN and binge eating/purging type of AN have a common (or similar) mechanism of prefrontal self-regulatory function representing patients’ impulsivity and eating behavior.

Meanwhile, the AN group showed no specific feature of task performance variables or prefrontal activation in the RPST_loss_ compared to the HC. This might be because restricting type of AN patients were included, who often show overly controlled behavioral style and less impulsive than binge eating/purging type of AN patients [9].

### Correlation Coefficients

Correlation analyses also revealed specific features of ED. First, both the AN and BN groups showed no significant correlation between RPST_loss_ task accuracy and the prefrontal [oxyHb], while the HC showed a moderate negative correlation like those of a previous study [25]. According to the aforementioned interpretations of the RPST_loss_, the correlation of the HC could be reworded to state that whoever could get a higher score needed less self-inhibition, while those with lower accuracy and felt difficulty required a higher prefrontal activity. Additionally, the lack of significance in these correlations in the AN and BN groups can be explained by inefficient self-regulatory function of their PFC. That is, their PFC might have reached upper limit of self-regulatory function and then run over a function-dependent activity control range seen in the HC, so even who could get a high score showed high activity.

Secondly, prefrontal activity during the RPST_loss_ task period correlated with some symptom scales. In the BN group, the correlation between the severity subscale of BITE and prefrontal activity suggested that the inefficient prefrontal self-regulatory function could associate with their symptoms. Interestingly, the restraint subscale of EDE-Q in the BN group showed positive correlation with the prefrontal activity. And at the same time, the severity subscale of BITE in the AN group positively correlated with the prefrontal activity, suggesting a correlation between a bulimic tendency and the functional inefficacy in prefrontal self-regulation, which may signal latent binge eating and purging behavior in the AN group. Thus, these findings suggest a common etiology of both binge eating/purging type of AN and BN with regard to patients’ prefrontal self-regulatory function.

There have been fewer functional neuroimaging studies in BN patients as compared with AN patients [18], [59], [60]. Our findings suggest a new potential investigational approach to understanding the pathophysiology of abnormal self-regulation of BN.

### Limitations

There are several methodological limitations in this study. The use of the two-channel NIRS, i.e. inexhaustive imaging method, could incur unavoidable misalignment of probes than areas of "ideal" activation peak pointed in the previous fMRI/PET/multi-channel NIRS researches thus might limit the symptom-related sensitivity. However, this choice with predetermined setup was not entirely a shortcoming but made this study stricter with respect to channel setting and analyzing process. Interchannel (not interprobe) distances are fixed with most of the available multi-channel probe sets, which can induce inappropriate measurements being not adjusted for individuals with different head sizes. There is another limitation that raw NIRS data should basically not be used for comparisons because of its fundamental that two measurements have different photo-pathlengths. Even if careful preprocessings and analyses are performed, NIRS data requires careful interpretation for comparisons between different channels, between different experimental days, or between individuals.

The number of subjects examined was relatively small for discussing group differences of brain functions. The limited number of participation might diminish the value of the controlling task performance parameters for BMI by not meeting prerequisites for analysis for covariance. Thus these analyses might not strictly rule out possible effects of malnutrition. The patient groups should consist of only drug-free patients to eliminate the possible effects of psychotropic medications known to modulate cerebral hemodynamic functions. We found no significant differences between patients with and without psychotropic medications, but the possibility of medication effects cannot be ruled out with such small sample. Some limitations were given by the use of NIRS, one of which is that brain regions observed with it are limited to narrow and superficial regions, but not localized. Furthermore, [oxyHb] data obtained from different pathlengths should not be compared between individuals or channels, and there is currently no standard method to standardize data. We attempted to avoid such limitations by using the z score.

### Conclusions

In conclusion of this preliminary study, our first hypothesis that the ED patients would have changes in cognitive abilities associated with self-regulation was partly confirmed in the BN group. The results of the BN group could support our second hypothesis, namely that the abnormal cognitive functions were accompanied by changes in prefrontal blood perfusion as a characteristic activation pattern, though the NIRS data showed no significant group difference directly. Third hypothesis was partly confirmed as that prefrontal activities associated with self-regulation in the RPST_loss_ of the AN and BN groups showed no significant correlation with the task accuracy, which the HC group showed a moderate negative correlation, and showed positive correlations with some symptom scales.
